# Bioclimatic and Land Use/Land Cover Factors as Determinants of Crabronidae (Hymenoptera) Community Structure in Yunnan, China

**DOI:** 10.3390/insects17010100

**Published:** 2026-01-15

**Authors:** Nawaz Haider Bashir, Muhammad Naeem, Qiang Li, Huanhuan Chen

**Affiliations:** 1 College of Biology and Food Engineering, Qujing Normal University, Qujing 655011, China; nawazhaider@mail.qjnu.edu.cn (N.H.B.); naeem@mail.qjnu.edu.cn (M.N.); 2 Yunnan International Joint Laboratory with South and Southeast Asia for the Integrated Development of Animal-Derived Anti-Thrombosis Chinese Medicine, Qujing Normal University, Qujing 655011, China; 3 Department of Entomology, College of Plant Protection, Yunnan Agricultural University, Kunming 650201, China; liqiangkm@126.com

**Keywords:** biogeographic zones, bioclimatic factors, remote sensing, GIS

## Abstract

Crabronid wasps (Hymenoptera: Crabronidae) are ecologically important predators that provide various ecological services by regulating the arthropod populations, enhancing soil processes through nesting, serving as sensitive indicators of habitat condition, and providing pollen transfer for plants. However, as other invertebrates face biodiversity threats, these wasps might be under threat from environmental changes, and we need to assess the biodiversity patterns of these wasps in Yunnan Province. Unfortunately, no information is currently available about the pattern and factors responsible for the assemblages of these wasps within our study region. This study provides the first province-level assessment of habitat suitability, species richness, assemblage structure, and environmental determinants for Crabronidae in Yunnan by integrating species distribution modeling (SDM), multivariate clustering, and ordination analyses. More than 50 species were studied to assess habitat suitability in Yunnan using MaxEnt. Model performance was robust (AUC > 0.7). Suitability patterns varied distinctly among regions. Species richness peaked in southern Yunnan, particularly in the counties of Jinghong, Mengla, Menghai, and Jiangcheng Hani & Yi. Land use/land cover (LULC) variables were the dominant predictors for 90% of species, whereas precipitation-related variables contributed most strongly to the remaining 10%. Ward’s hierarchical clustering grouped the 125 counties into three community assemblage zones, with Zone III comprising the most significant area. A unique species composition was found within a particular zone, and clear separation among zones based on environmental variation was supported by Principal Component Analysis (PCA), which explained more than 70% variability among zones. Furthermore, Canonical Correspondence Analysis (CCA) indicated that both LULC and climatic factors shaped community structure assemblages, with axes 1 and 2 explaining 70% of variance (*p* = 0.001). The most relevant key factors in each zone were precipitation variables (bio12, bio14, bio17), which were dominant in Zone I; for Zone II, temperature and vegetation variables were most important; and urban, wetland, and water variables were most important in Zone III.

## Simple Summary

Many ecologically and economically important species of Crabronidae wasps (Hymenoptera) perform hunting of soft-bodied insect pests and pollen transformation services. Because of these highly beneficial economic services, Crabronidae deserve to be studied. This paper investigates the impact of environmental factors on the community structure and distribution patterns of these wasps in Yunnan Province, China. The Yunnan Province is an ideal place for this study as it is a biodiversity hotspot within China due to its climatic and topographic conditions. Our results suggest that environmental factors influenced the shape and community assemblages of Crabronidae species in Yunnan. In addition, different areas of the provinces support different species and this pattern is strongly associated with environmental factors. The land use/land cover (LULC) factors played a primary role in most species’ assemblages, whereas bioclimatic factors played a secondary role. Finally, we indicate that the wasp species are most sensitive to environmental changes, including climatic and land-cover changes, emphasizing the need for effective habitat protection measures. The information obtained from this study provides a baseline that can help conservation planning, monitor future challenges and support efforts to maintain healthy wasp communities.

## 1. Introduction

Crabronid wasps (Crabronidae) contribute to biodiversity by building nests within ecosystems, regulating insect populations, and providing pollination services [1,2]. Through their nesting activities, these wasps influence soil health and participate in complex mutualistic interactions with microbial symbionts [3]. They play a significant role in ecological balance by reducing pest abundance, supporting food web dynamics, and serving as natural indicators of habitat quality [4]. However, insect diversity is broadly threatened by various factors [5,6,7], and crabronid wasps also face similar challenges. Recent studies indicate that insect decline is not confined to one region, but is a global phenomenon affecting pollination, nutrient cycle, and food webs [8,9].

Community assemblage is associated with species co-occurrence patterns and community structure, driven by interactions between organisms and their environment [10]. Assemblage patterns help us understand how communities respond to climate change, disturbances, and habitat fragmentation, as well as why similar habitats can host different communities across regions [11]. These interacting species (assemblages) can be represented graphically or spatially in a particular region using mapping, which helps study patterns of interactions [12] and local ecological processes [13].

In community assemblages, multiple factors may affect community composition, such as land use/land cover (LULC), biotic, climatic, and biogeographic factors [11]. Even for similar species, these factors may contribute differently to community assemblages from region to region [14]. For instance, regional differences in LULC factors lead to distinct assemblage responses whose effect on dragonfly and damselfly species varies among zones [15]. Another study integrated long-term arthropod distribution data with climate and land-use change variables and found that rapid climatic and anthropogenic changes destabilize biodiversity and community assemblages [16]. The effect of climate change and LULC drivers was not studied in Crabronidae.

Yunnan is situated at the intersection of three global biodiversity hotspots (eastern Himalayas, southwest China mountains, and Indo-Burma region) and includes more than 50% of all species reported from China [17]. Recent studies also indicate that many new species and country records still have to be discovered there [18,19,20]. In the Yunnan Province, research on crabronid species has primarily focused on taxonomic work [21,22,23,24]. The primary focus of this work is to map the spatial patterns of crabronid species assemblages across the Yunnan Province and to determine the relative contributions of bioclimatic and land-use/land-cover (LULC) factors. According to our hypotheses, both climatic and LULC factors influence the community structure of crabronid species in Yunnan. Specifically, we expect climate variables to be the main drivers at broader spatial scales, whereas LULC factors will be more influential in shaping local assemblage patterns.

## 2. Materials and Methods

### 2.1. Study Site and Data Collection

**Figure 1:**
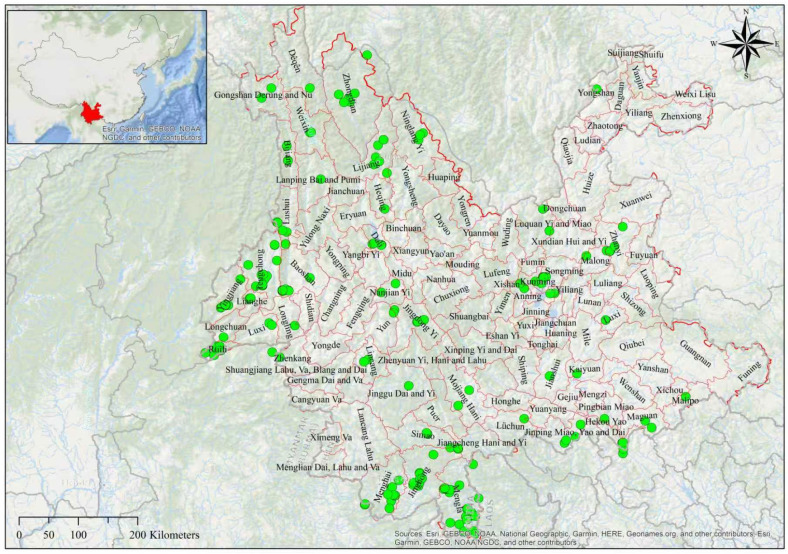
The administrative boundary of Yunnan Province, with its 125 counties shown on a shaded relief map. Green dots are the collection sites of 53 Crabronidae species.

The Yunnan Province, located in southwestern China, is divided into 125 counties and extends between 21°09′–29°15′ N and 97°32′–106°12′ E (Figure 1). We collected Crabronidae from different locations of Qujing (25°31′34″ N, 103°45′06″ E; 25°31′38″ N, 103°44′51″ E; 25°31′22″ N, 103°44′41″ E; and 25°53′47″ N, 103°56′39″ E) and Shangri-La (27°54′07″ N, 99°38′20″ E) during 2024–2025. The other Yunnan distribution records of Crabronidae were taken from the Catalog of Sphecidae [25] and the recent literature [22,24,26,27,28,29].

In order to assess the response of Crabronidae to bioclimatic and land-use/land-cover (LULC) factors, a data set of 112 crabronid species across 241 collection sites was analyzed. Spatial rarefaction was applied to reduce autocorrelation in the data, and only records for each species with an area greater than 1 km^2^ were retained. Additionally, duplicate collections were carefully removed, leaving us with 151 collection sites and 53 species, providing a more precise and accurate understanding (Figure 1).

The bioclimatic analysis for the current period was performed using data obtained from www.worldclim.org (accessed on 22 October 2025). Initially, 19 bioclimatic variables were considered; later, we removed highly correlated variables from the analysis using Pearson’s correlation, and retained nine non-correlated variables (r < 0.9): bio2, bio3, bio4, bio7, bio10, bio12, bio14, bio15, and bio17. To minimize redundancy, this threshold is widely used to eliminate highly correlated variables, and the filtering process follows standard practices for species distribution modeling [30]. A spatial resolution of approximately one km^2^ was obtained for these variables, and Pearson correlation coefficients were determined using ArcGIS v 10.8.

LULC (land use land cover) data were obtained from the MCD12C1.061 MODIS Land Cover Type Yearly Global dataset accessed via Google Earth Engine for 2023. The Majority_Land_Cover_Type_1 band was used to identify 17 land cover classes, which were then grouped into five categories: urban, barren, wetland, water, and vegetation, including grasslands, forests, shrublands, savannas, and croplands.

### 2.2. Habitat Suitability Assessment and Statistical Analysis

A MaxEnt-based species distribution modeling approach was used in MaxEnt software (version 3.4.4) with five replicates per species to assess habitat suitability ranges for crabronid species [31]. The accuracy of the models was evaluated by analyzing the area under the curve (AUC) of the receiver operating characteristic (ROC) statistic [31].

In each county, habitat suitability ranges were calculated for all crabronid species, and the average of habitat suitability values was summed across all pixels. We created a helpful matrix that shows the relationship between different species and counties (sites). In this matrix, each row corresponds to a specific county (or site), and each column represents a particular species. This setup makes it easier to understand how species are distributed across various locations (Table S1).

The pixels having values > 0.5 were considered suitable, and those having values less than 0.5 were considered unsuitable. Using MaxEnt regularized training gain, percent contribution, permutation importance, and jackknife tests were conducted to estimate variable importance. Additionally, response curves were analyzed to verify plausible ecological relationships. Species predicted by a predictor are considered dominant when a prediction is placed at the top of a permutation importance list, and when it provided either the most considerable percent contribution or the most significant reduction in gain when it was omitted in the jackknife test [32,33].

To understand environmental variability and show the structure of gradients in the study area, Principal Component Analysis (PCA) was performed [34]. Ward’s hierarchical clustering algorithm was used with Euclidean distance to cluster 125 counties in Yunnan Province to analyze crabronid species assemblage and patterns. The number of clusters (k) for k = 2 to 10 was determined by evaluating the average silhouette width, Davies–Bouldin index, and Calinski–Harabasz index [35,36,37]. Based on habitat suitability values, a potential species richness was calculated for each county, and a Canonical Correspondence Analysis (CCA) was performed to examine the influence of individual variables on clustering or spatial patterns of crabronid species within Yunnan Province. In our study, CCA was conducted using habitat suitability values for 53 crabronid species as community input. The CCA is commonly applied to abundance data; however, we applied it to suitability values due to the incompleteness of the empirical abundance data. A similar approach has already been used in other macroecological studies [38,39]. In the CCA, the significance of the axes was assessed using 999 permutations. The analyses were conducted using Python 3.12.4. Pandas and NumPy were used to manipulate data and perform numerical computations.

## 3. Results

### 3.1. Mapping of Crabronidae Community in Yunnan

**Figure 2:**
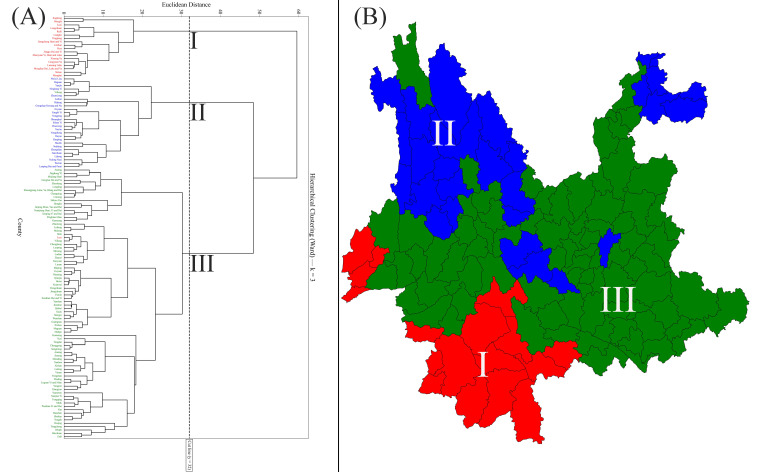
Crabronidae community zones (I, II, and III) in Yunnan based on Ward’s agglomerative cluster analysis: (**A**) Three distinct zones were identified using Euclidean distance and a phenon line of 30.2; (**B**) The spatial distribution of three zones in Yunnan Province.

In Yunnan Province, the counties were divided into three zones, ranging from 18 to 80 counties in cluster size, as Ward’s agglomerative cluster analysis revealed that k = 3 gave the strongest solution (Calinski–Harabasz = 48.76, Davies–Bouldin = 1.19, and silhouette = 0.30) (Table S2; Figure 2). Most counties were assigned to Zone III (64.0%), followed by Zone II (21.6%), and Zone I (14.4%) (Figure 2A). Figure 2B illustrates the spatial distribution of these zones.

**Figure 3:**
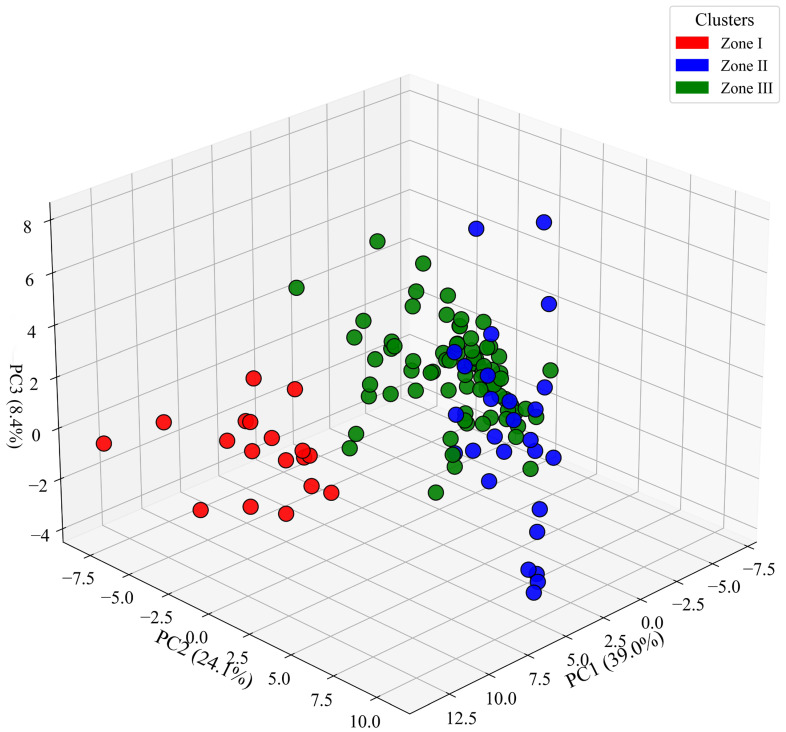
The cluster patterns of 125 counties of Yunnan Province, China, show the zones I, II, and III based on Principal Component Analysis (PCA). Circular points represent counties within Yunnan Province.

We conducted Principal Component Analysis (PCA) to better understand zonation patterns in relation to environmental variability across counties (Figure 3). Three PCA axes together captured 71.5% of the variation in the Crabronidae community across 125 counties in Yunnan Province. Specifically, PCA1 explained 39%, PCA2 explained 24.1%, and PCA3 explained 8.4% of the observed differences (Figure 3).

### 3.2. Crabronidae Community Assemblages and Their Environmental Factors in Yunnan Province, China

**Figure 4:**
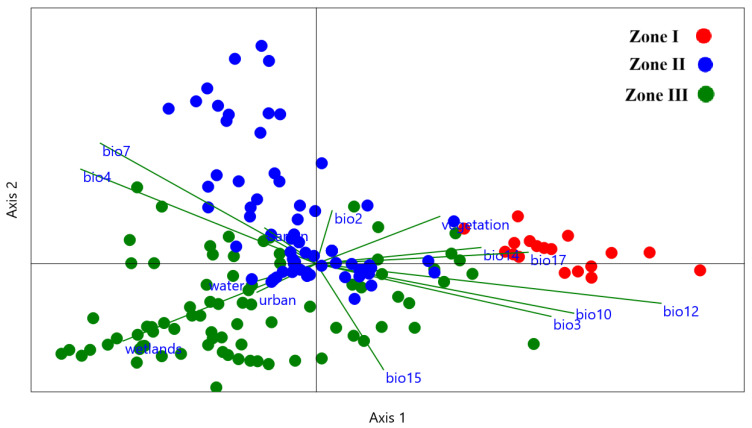
Crabronidae community assemblages in Yunnan Province and their environmental factors illustrated by Canonical Correspondence Analysis (CCA). On the CCA plot, points represent county locations based on the scores for axes 1 and 2. Arrows’ length and direction represent the significance and impact of each environmental variable on crabronid species across the counties. The temperature-related bioclimatic variables are bio2 (Mean diurnal range), bio3 (Isothermality), bio4 (Temperature seasonality), bio7 (Temperature annual range), and bio10 (Mean temperature of warmest quarter), while precipitation-related bioclimatic variables are, bio12 (Annual precipitation), bio14 (Precipitation of driest month), bio15 (Precipitation seasonality), and bio17 (Precipitation of driest quarter) (www.worldclim.org).

Using Canonical Correspondence Analysis (CCA), we analyzed crabronid community assemblages across various locations in Yunnan Province in response to different environmental factors (Figure 4). Based on their eigenvalues, axes 1 and 2 explain 40.28% and 29.75% of the variance, respectively. These two axes together explain 70% of the overall variability, validated by a permutation “*p*” value of 0.001. Consequently, the identified community assemblage trends (Figure 2) show significant influences from the 14 environmental factors (Figure 4). The primary variables in Zone I were bio12, bio14, and bio17. In Zone II, bio2, barren, bio7, and vegetation were identified as the leading environmental factors. Urban, wetland, water, and bio15 were strongly linked to crabronid community patterns in Zone III (Figure 4). Environmental factors were positively and negatively correlated with axes 1 and 2 (Table 1). In total, 8 of the 14 environmental factors had strong positive correlations with Axis 1, with the highest correlation found for bio12 (0.81), followed by bio10 (0.60), bio3 (0.55), and bio17 (0.49). Axis 2 displayed positive correlations with half (50%) of the environmental factors, particularly with bio7 (0.63), followed by bio4 (0.49), bio2 (0.28), and vegetation (0.25) (Table 1).

### 3.3. Habitat Suitability of Crabronidae Community Assemblages and Contributing Variables

**Figure 5:**
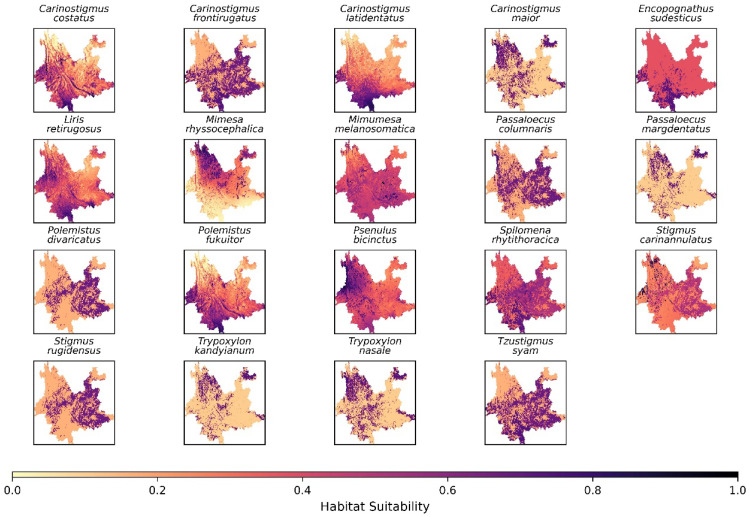
Spatial distribution of habitat suitability of some representative species of different zones in Yunnan Province, China. Light yellow color represents the lowest habitat suitability, whereas darker indicates the highest suitability.

The mapping of community assemblages of crabronid wasps was based on habitat suitability modeling. We used 25% of the data for training and the remaining 75% for testing the model. For both training and testing datasets, MaxEnt’s species distribution models demonstrated area under the curve (AUC) values more than 0.7, which indicates reliable performance in habitat suitability. All species were assigned habitat suitability values ranging between 0 and 1 (Figure 5 and Table S3).

**Figure 6:**
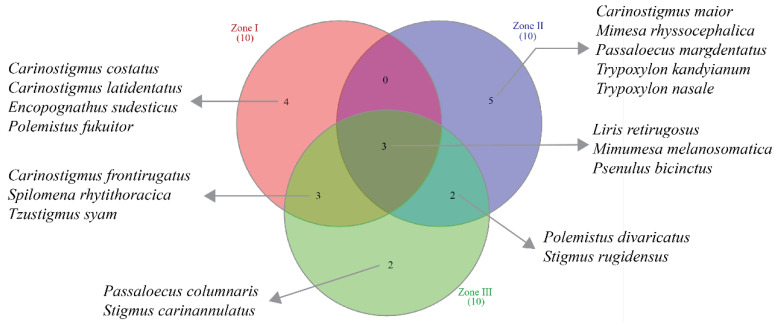
Venn diagram showing the unique and shared species having the most habitat suitability within three zones of Yunnan Province, China.

Different species exhibit high habitat suitability across the different zones of Yunnan Province. For example, the top 10 species showing highest habitat suitability within zone I are *Carinostigmus costatus* Krombein (1984), *C. frontirugatus* Bashir and Ma (2020), *C. latidentatus* Bashir and Ma (2020), *Encopognathus sudesticus* Leclercq (1977), *Liris retirugosus* Li, Cai, and Li (2009), *Mimumesa melanosomatica* Ma and Li (2009), *Polemistus fukuitor* Tsuneki (1992), *Psenulus bicinctus* Turner (1912), *Spilomena rhytithoracica* Li and He (1998), and *Tzustigmus syam* Finnamore (1995). The top 10 species of zone II are *Carinostigmus maior* (Maidl, 1925), *Liris retirugosus* Li, Cai, and Li (2009), *Mimesa rhyssocephalica* Ma, Li, and Chen (2008), *Mimumesa melanosomatica* Ma and Li (2009), *Passaloecus margdentatus* Li and Ma (2024), *Polemistus divaricatus* Ma and Li (2020), *Psenulus bicinctus* Turner (1912), *Stigmus rugidensus* Li and Ma (2024), *Trypoxylon kandyianum* Tsuneki (1979), and *T. nasale* Tsuneki (1979). Similarly, the species of zone III showing highest habitat suitability are *Carinostigmus frontirugatus* Bashir and Ma (2020), *Liris retirugosus* Li, Cai, and Li (2009)*, Mimumesa melanosomatica* Ma and Li (2009), *Passaloecus columnaris* Ma and Li (2012)*, Polemistus divaricatus* Ma and Li (2020), *Psenulus bicinctus* Turner (1912), *Spilomena rhytithoracica* Li and He (1998), *Stigmus carinannulatus* Li and Ma (2024), *Stigmus rugidensus* Li and Ma (2024), and *Tzustigmus syam* Finnamore (1995). Although the above top 10 species of each zone also share habitat suitability within other zones, some unique species show habitat suitability within particular zones (Figure 6).

**Figure 7:**
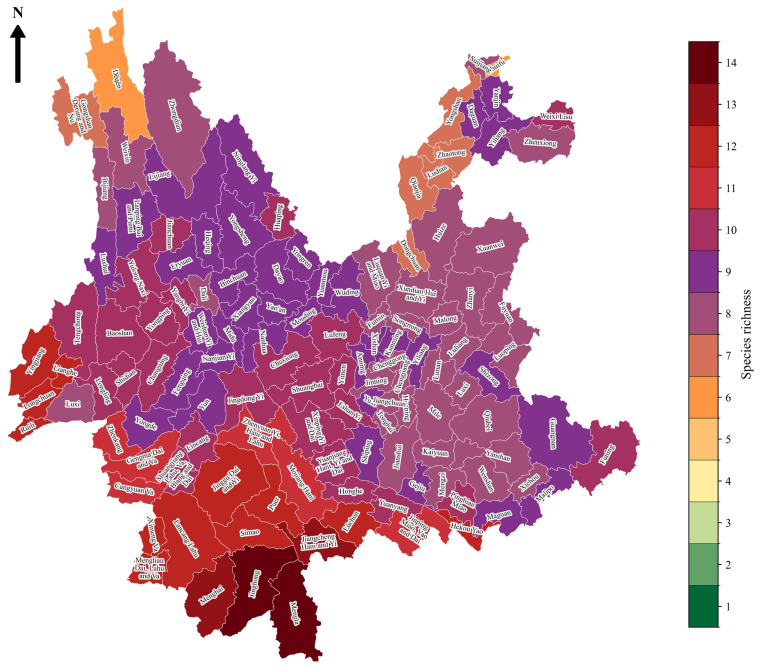
The species richness pattern of Crabronidae within each county of Yunnan Province, China.

In the Yunnan Province, 90% of crabronid species demonstrate LULC as the predominant environmental factor in spatial distribution modeling; the remaining 10% were influenced by precipitation-related variables and LULC (Figure S1). The highest species richness, reaching up to 14 species, was observed in Jinghong and Mengla counties, followed by Menghai and Jiangcheng Hani & Yi counties, each with 13 species. All these counties are situated in the southern region of the Yunnan Province (Figure 7).

## 4. Discussion

For effective conservation, it is crucial to study the community assemblages of particular species at a local scale [40,41]. Hence, we studied the community assemblages of crabronid wasps and the associated factors shaping these assemblages in the Yunnan Province. The highest species richness (*n* = 14) was detected in the southern region of the province (Figure 7). Covering the land with abundant vegetation and using natural resources have been linked to higher species richness. The southern counties of Yunnan, which show rich biodiversity, might have greater vegetation diversity (Figure 7) [42,43].

Geographical sampling bias may partly explain the higher species richness in Zone I of the southern region of Yunnan, where occurrence data were more abundant (Figure 1). Although we relied on SDM suitability predictions rather than raw records to estimate richness, residual bias remains in the input data. In the same way, the relationship between higher vegetation cover (greater plant density and land cover) and increased predicted county richness was observed descriptively rather than formally assessed. Nevertheless, future studies using standardized survey data and statistical models could confirm the factors of these patterns.

Our findings reveal that the distribution of crabronid wasps in Yunnan is most strongly influenced by land-use and land-cover (LULC) patterns. In our models, LULC emerged as the highest-contributing predictor, surpassing bioclimatic variables. This indicates that crabronid wasps respond more sensitively to habitat structure, landscape configuration, and anthropogenic modification than to broad climatic gradients. Previous research on Hymenopteran pollinators, particularly bumblebees, has consistently shown temperature as the key factor of distribution and activity patterns [40,44,45,46]. However, very few studies have incorporated detailed LULC variables into models of the distribution of Crabronidae or related solitary wasps, leaving a gap in understanding how habitat conversion influences these species. Our findings strongly suggest that habitat change such as expanding cropland, urban development, and shrinking semi-natural areas poses a more urgent threat to crabronid wasps than climate factors. This underscores the ecological value of preserving structurally diverse landscapes to sustain these vital pollinators and biocontrol agents.

Based on the 3D PCA, three distinct zones have been identified in all counties (125) of the Yunnan Province (Figure 3). By combining these three main components, 71.5% of the data’s variability was captured, highlighting the most important mechanisms or zonation patterns [47]. The application of multivariate PCA techniques to assess biogeographic regionalization patterns has already been successfully demonstrated in the study of bumblebee (genus *Bombus*) species assemblage patterns throughout Yunnan and other regions [39,48,49]. The precise identification of three community assemblage zones in Yunnan suggests that the environmental factors included in our analysis successfully accounted for the spatial patterns observed in the community.

An analysis of Ward’s agglomerative clustering explains the landscape’s spatial heterogeneity and the influence of environmental gradients on community structure [50,51] (Figure 2). Using Ward’s hierarchical clustering and PCA methods, Yunnan was delineated into three zones, in alignment with other regional studies. As shown by a recent study in Gansu and Yunnan Provinces (China), distinct biogeographic regions were identified [48]. Different environmental factors shaped these regions. Using multivariate methods to define zonation patterns of crabronid wasps shows that our analysis is valid. Statistically, these methods effectively minimize variance within clusters, as originally described by Ward [52].

Based on 14 environmental factors, our CCA results provide compelling evidence of crabronid wasp community assemblages in Yunnan. Based on the permutation test results, the first two axes explained over 70% variance, indicating the variables chosen for this study are significant (*p* = 0.001) influences on Yunnan community assemblages [53] (Figure 4). There are distinctive zones corresponding to different factors, particularly the LULC and bioclimatic variables related to community assemblages. Species distribution patterns are strongly influenced by bioclimatic variables, as reported in previous studies [54].

Due to our modeling framework, we used only one MaxEnt replicate per species. This approach prevents us from directly estimating uncertainty, such as standard errors or confidence intervals, around suitability measures. Although our models showed good performance (AUC > 0.7), we recognize that future research should include resampling or replication to quantify prediction uncertainty, especially in conservation planning. Moreover, CCA input values were derived from SDMs rather than occurrence and abundance data. A substitution like this may influence the strength of the species-environment relationship inferred by this process. There are no standardized community-level surveys available for all counties in Yunnan. As a result, SDM proxies provide an applied alternative for large-scale biogeographic studies [38]. The species richness maps we generate are based on aggregated results from suitability analyses rather than direct observations, so they may be subject to pseudo-replication. For comparative purposes, these maps are valuable in identifying potential patterns of richness, but they should not be considered absolute measures of diversity.

We focused our analysis on environmental variables without explicitly including biotic interactions (e.g., floral resource availability, competition), anthropogenic pressures, and elevation, which may influence the distribution of Crabronidae in the Yunnan Province as well. Because the province lacks standardized data on these factors, they were excluded. Yunnan lies within the Indo-Burma biodiversity hotspot, but we did not compare its assemblage patterns with those of other hotspots. The framework we developed could be applied to future climate scenarios and cross-hotspot comparisons, making it more useful for conservation. Furthermore, MaxEnt parameters were not sensitivity analyzed (regularization multipliers), but future work could test predictability by varying model settings and applying model ensembles. However, we found that our study provided a valuable baseline assessment of Crabronidae assemblages based on the current environment.

The results of this study showed that the wasp community of Crabronidae in Yunnan was shaped not only by broader environmental gradients but also by finely tuned local environmental conditions [55]. Based on the clear zonation pattern observed in our study, it is imperative that we implement targeted conservation strategies that account for both regional environmental drivers and local habitat characteristics in Yunnan Province to ensure the survival of particular species in the region. In addition, future studies should use more detailed habitat data and climate models to predict how these assemblages may change as the climate warms.

## 5. Conclusions

In mapping assemblages of Crabronidae, fine-tuned local habitat conditions determine species distributions and assemblage patterns. The patterns of distribution and assemblages of more than 50 Crabronidae wasp species within the three distinct zones of Yunnan Province indicated that a particular zone is associated with distinct wasp species. CCA further demonstrated that precipitation, vegetation, wetlands, and temperature seasonality significantly shape community structure. Together, these findings indicate that interactions between broad-scale climate gradients and local habitat conditions drive crabronid assemblage patterns. The spatial framework developed here provides crucial guidance for biodiversity conservation planning and monitoring. Future work should integrate finer-scale habitat data and long-term surveys to better understand the environmental effects on Crabronidae communities in this region.

## Figures and Tables

**Table 1:** CCA (Canonical Correspondence Analysis) relationship between environmental factors and axes.

Environmental Factors	Axis 1 (CCA Plot)	Axis 2 (CCA Plot)
bio10	0.60	−0.26
bio12	0.81	−0.21
bio14	0.38	0.08
bio15	0.16	−0.55
bio17	0.49	0.06
bio2	0.04	0.28
bio3	0.55	−0.27
bio4	−0.55	0.49
bio7	−0.50	0.63
water	−0.16	−0.11
vegetation	0.29	0.25
wetlands	−0.45	−0.40

## Data Availability

The original contributions presented in this study are included in the article/supplementary material. Further inquiries can be directed to the corresponding author.

## Supplementary Materials

The following supporting information can be downloaded at: https://www.mdpi.com/article/10.3390/insects17010100/s1, Table S1. Habitat suitability ranges for Crabronidae (each row represents a specific county and each column represents a specific species). Table S2. Validation of hierarchical clustering on county × suitability dataset (k = 2 to k = 10). Table S3. Community assemblages of Crabronidae in different zones in Yunnan Province, China. Figure S1. Contribution of different variables in the spatial distribution of Crabronidae species within Yunnan, China.
