# Supplementary material for: Bioclimatic and Land Use/Land Cover Factors as Determinants of Crabronidae (Hymenoptera) Community Structure in Yunnan, China
Source: Insects. 2026 Jan 15;17(1):100. doi: 10.3390/insects17010100 (PMC12842201; doi:10.3390/insects17010100)
Supplement: Supplementary file 1 [file insects-17-00100-s001.zip › Supplementary material/Figure S1.pdf]

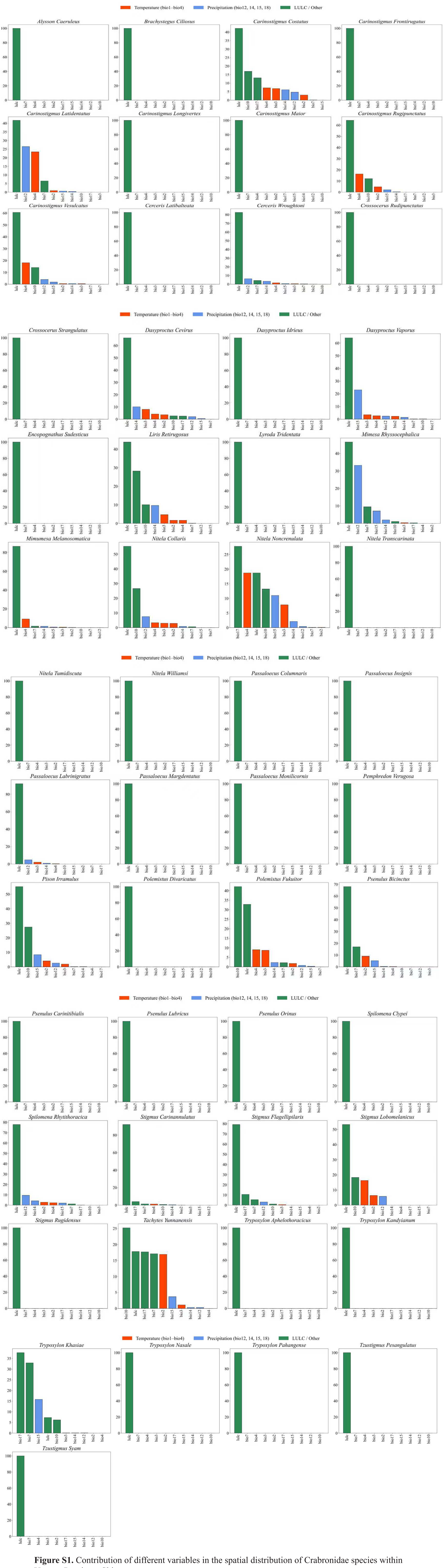

Figure S1. Contribution of different variables in the spatial distribution of Crabronidae species within Yunnan Province, China.
