# Supplementary material for: Bioclimatic and Land Use/Land Cover Factors as Determinants of Crabronidae (Hymenoptera) Community Structure in Yunnan, China
Source: Insects. 2026 Jan 15;17(1):100. doi: 10.3390/insects17010100 (PMC12842201; doi:10.3390/insects17010100)
Supplement: Supplementary file 1 [file insects-17-00100-s001.zip › Supplementary material/Table S2.pdf]

Table S2. Validation of hierarchical clustering on county × suitability dataset (k = 2 to k = 10)

k	Calinski–Harabasz	Davies–Bouldin	Silhouette
2	44.8774	0.9998	0.3583
**3**	**48.7591**	**1.1940**	**0.3047**
4	42.5061	1.3882	0.2397
5	37.3027	1.6870	0.1957
6	34.4883	1.5993	0.1910
7	31.9632	1.4816	0.1991
8	30.3072	1.3293	0.1983
9	29.2983	1.3134	0.1961
10	28.8121	1.2493	0.2013
